# Detection of Occult Acute Kidney Injury in Glucose-6-Phosphate Dehydrogenase Deficiency Anemia

**DOI:** 10.4084/MJHID.2016.038

**Published:** 2016-08-20

**Authors:** Gehan Lotfy Abdel Hakeem, Emad Allam Abdel Naeem, Salwa Hussein Swelam, Laila El Morsi Aboul Fotoh, Abdel Azeem Mohamed El Mazary, Ashraf Mohamed Abdel Fadil, Asmaa Hosny Abdel Hafez

**Affiliations:** 1 Pediatric Department, Minia University, Egypt; 2 Clinical Pathology Department, Minia University, Egypt

## Abstract

**Background:**

Glucose-6-phosphate dehydrogenase (G6PD) deficiency anemia is associated with intravascular hemolysis. The freely filtered hemoglobin can damage the kidney. We aimed to assess any subclinical renal injury in G6PD children.

**Methods:**

Sixty children were included. Thirty G6PD deficiency anemia children were enrolled during the acute hemolytic crisis and after the hemolytic episode had elapsed. Another thirty healthy children were included as controls. Serum cystatin C, creatinine levels, and urinary albumin/creatinine (A/C) ratio were measured, and the glomerular filtration rate (GFR) was calculated.

**Results:**

Significantly higher urinary A/C ratio (*p*=0.001,0.002 respectively) and lower GFR (*p*=0.001 for both) were found during hemolysis and after the hemolytic episode compared to the controls. Also, significant higher serum cystatin C (*p*=0.001), creatinine (*p*=0.05) and A/C (*p*= 0.001) ratio and insignificant lower GFR (*p*=0.3) during acute hemolytic crisis compared to the same children after the hemolytic episode subsided.

**Conclusions:**

G6PD deficiency anemia is associated with a variable degree of acute renal injury during acute hemolytic episodes which may persist after elapsing of the hemolytic crises.

## Introduction

Glucose-6-phosphate dehydrogenase (G6PD) deficiency anemia is an X-linked recessive hereditary disease where the enzyme G6PD is deficient.^1^ G6PD is the key regulatory enzyme in the hexose monophosphate shunt with the production of nicotinamide adenine dinucleotide (NADPH) that is required for protection against oxidative damages.^2^ Increased oxidative stress has been observed in many diseases including those related to renal damage.^3^ There is a higher prevalence of the G6PD deficiency in children with unexplained chronic kidney disease assuming that G6PD deficiency anemia may play an important role in the pathogenesis of chronic kidney disease.^4^ In G6PD deficiency, massive intravascular hemolysis can cause acute renal failure, and acute tubular necrosis might complicate the severe hemolytic episode*.*^5^ Accurate renal function measurements are important for diagnosis, treatment, and prevention of more severe renal damage. Serum Creatinine (Cr) is the most commonly used indicator of renal function, but its measurement suffers from a variety of analytical interferences and significant standardization problems.^6^ Cystatin C is a low-molecular-weight protein freely filtered by the glomeruli. Its serum concentration is less dependent on extra renal factors than in the case of Creatinine.^7^

The aim of this study was to detect occult acute kidney injury in glucose six phosphate dehydrogenase anemic children with apparently normal kidney function during and after termination of the acute hemolytic crises by assaying the glomerular filtration rate (GFR), serum creatinine and urinary albumin/creatinine (A/C) ratio.

## Materials and Methods

This case-control study was carried out between March and August 2014. Sixty children were enrolled in this study which involved thirty known glucose-6-phosphate dehydrogenase deficiency (G6PD) anemia children (group I) were included and thirty healthy age and sex matched control group (II). The G6PD assay was based on the modified Beutler fluorescent spot test^9^ using the G6PD screening test (Kimia Pagouhan lot no 90607, Iran). The G-6-PD deficiency was defined as any G-6-PD value <3.4 U/gHb. This cut-off was adopted for both males and females. In the present series, 40% of affected children were females. This percentage is larger if compared with previous Egyptian studies, but very similar to that found in malaria endemic areas in which the ratio males/females is about 1.6/1.^10^–^13^ Of course, this group included heterozygotes phenotypically affected because of the phenomenon of Lyonization^14^ and homozygous. The WHO imposed a fixed threshold of 10% of normal value to consider heterozygotes being phenotypically deficient, as did the most recent study by Nkhoma et al.^11^ However, across the predicted national predictions, a median proportion of 26.4% (IQR: 25.2–27.6) expected heterozygotes were predicted to be phenotypically deficient.^12^ The homozygous quote is dependent on the frequency of affected gene in the regions and by marriage consanguinity.^13^ Although G6PD deficiency is frequently considered to be rare in females, it is clear from the assembled database and derived modeled population estimates that in many areas an important proportion of females will also be affected.^15^ The aim of our study was not epidemiological, and this large proportion of female could be casual and not reflect the real situation. However, it suggests further studies on a wider scale of patients, since the complexity of ethnic populations in Egypt.

For all patients with low G6PD level, DNA was extracted by a phenol-chloroform based method.^16^ The extracted DNA was screened sequentially for four G6PD deficient mutations namely G6PD Mediterranean (563 C→T), G6PD Chatham (1003 G→A), G6PD Cosenza (1376 G→C), G6PD A- (202 G→A) mutations using polymerase chain reaction/restriction fragment length polymorphism (PCR/RFLP) based method using standard column kits (Favorgen Biotech Corp., Taiwan). Twenty-seven (90 %) were G6PD-Mediterranean, 2 (6.7 %) were G6PD-Chatham, another 1 (3.3 %) were G6PD-A.

Patients of Group I were furtherly classified into two subgroups according to the presentation:

- Group Ia Included the thirty known G6PD anemic children during acute hemolytic crises just before a scheduled transfusion. Transfusion therapy had been given immediately after sampling as scheduled for acute hemolysis.
- Group Ib: included the same patients included in group Ia one month after the hemolytic crises had elapsed (proved by normal hemoglobin level, normal reticulocyte count, no urobilinogen in clear colored urine).

All patients were selected from the pediatric hematology emergency department (group Ia) and the pediatric hematology outpatient clinic during follow-up (group Ib) in Minia children’s University Hospital. The controls were selected from healthy school children after exclusion of G6PD anemia (by complete blood count and quantitative G6PD enzyme assay by the same method as involved patients) and renal disease (urinalysis blood urea, serum creatinine and urinary A/C ratio). The blood samples from the control group were taken at their schools.

The consent form was taken before blood sampling. Moreover, all patients and controls included were subjected to:

### History

Name, age, sex, residence, family history, and history of triggering factors for hemolysis.

Twenty-five (88.3%) of the involved patients had fava bean induced hemolysis, 3 (10%) patients had acute hemolysis following a viral upper respiratory tract infection and 2 (6.7%) patients had drug induced hemolysis (one patient received anti-diarrheal drug containing nitrofurantoin, and the other patient received ibuprofen, but both drugs were within the therapeutic levels).

### Examination

General examination, anthropometric measures which were plotted on percentile growth charts, vital data as well as an examination of chest, heart and abdomen.

### Exclusion criteria

Children with a history of renal disease or renal transplantation before admission, history of proven congenital renal anomalies, known autoimmune, renal disease, children with any malignancy, children with known other hematological diseases (Sickle cell anemia) and children with known metabolic diseases were excluded.

### Laboratory Investigation

performed to all involved subjects (group Ia, Ib, and II) Complete Blood Count (CBC), serum creatinine, indirect serum bilirubin, serum cystatin C, urinary A/C ratio.

Acute hemolytic crises are diagnosed clinically by acute pallor, jaundice, and dark urine. Normocytic normochromic anemia and reticulocytosis by CBC, indirect hyperbilirubinemia with normal liver enzymes.

Cystatin C-based GFR is calculated by Le Bricon formula: = [(78) × (1/cystatin C)] + 4^17^

Urinary albumin to creatinine ratio (A/C) using the National Kidney Foundation’s level recommendations as a reference (female <3.5 mg/mmol, male <2.5 mg/mmol)^18^

The study was conducted according to the Declaration of Helsinki and was approved by Minia Faculty of Medicine institutional review board.

### Sampling

Three Venous blood samples were collected from both patients and controls under complete aseptic conditions and divided as follow:

- About 2 ml of blood in a K3-EDTA anticoagulated tubes for complete blood count, peripheral smear as well as flow cytometric analysis and analyzed within 24 hours.
- About 3ml of blood in a plain tube without any anticoagulant left to clot and centrifuged at 2000 revolutions per minute (rpm) for 5 minutes. The serum was then separated, aliquoted and stored at − 20°C till used for serum cystatin C assay.
- About 3ml of blood in a plain tube without any anticoagulant left to clot and centrifuged at 2000 revolutions per minute (rpm) for 5 minutes. The serum was then separated and used for serum creatinine and indirect bilirubin assay.

Urine samples were collected for assaying A/C ratio.

### Methods of assay

Complete blood count was performed using automated blood counter (Sysmex KX-21N). Serum cystatin C (quantified by a turbid metric method (Roche Diagnostics, Indianapolis, IN), initial serum creatinine levels (were assayed by enzyme immune assay), indirect serum bilirubin using fully automated chemical auto-analyzer Dimension-ES, USA. Urinary A/C ratio using a turbid metric method at Quest Diagnostics Laboratories (San Juan Capistrano, CA). Creatinine was measured by Mind rays BS 300 chemical analyzer.

### Statistical Analysis

The SPSS (Statistical Package for the Social Sciences) statistical software suite, version 16.0, was used for all statistical analyses (SPSS Inc, Chicago, IL, USA). Data with normal distribution were expressed as the mean values ±SD and were assessed by paired Student’s *t*-test to evaluate inter-group (group Ia *versus* group Ib) differences. Data with skewed distribution were expressed as medians with corresponding interquartile ranges and were assessed by the Wilcoxon test for the inter-group comparisons. Differences between categorical variables were analyzed using Chi Square test. The relationship between serum cystatin C and clinical and laboratory variables were evaluated by partial correlation using Pearson test. A two-tailed *P* value <0.05 indicated statistical significance.

## Results

Group I included thirty children with an age ranging from 5 to 90 months. 18 (60%) of them were males and 12 (40%) were females^12^ while group II age ranged from 2-to 95 months, 18 (60%) were male, and 12 (40%) were female. (Table 1)

**Figure 1:**
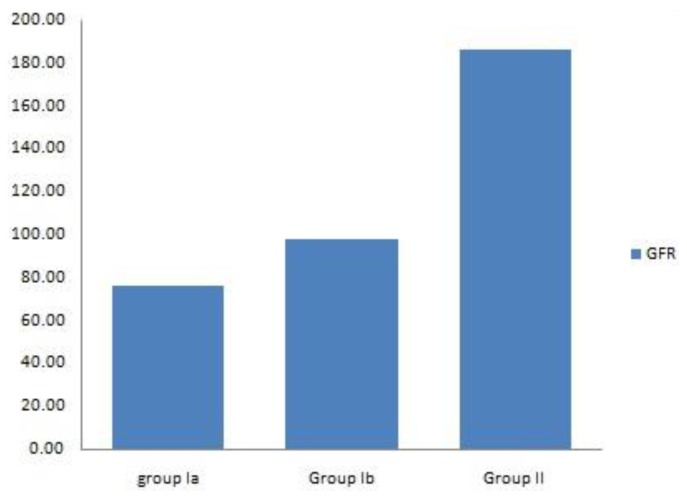
Comparison between studied groups regarding GFR.

**Figure 2:**
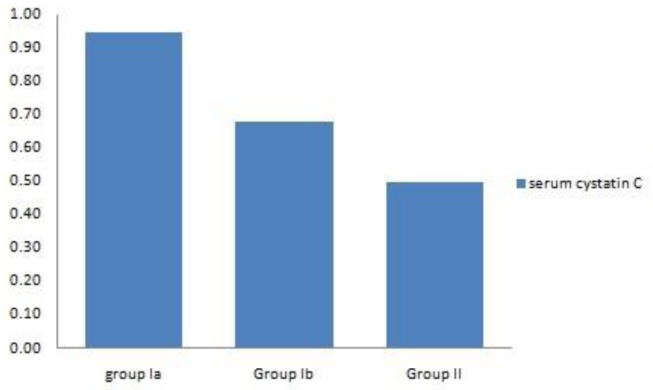
Comparison between studied groups regarding serum cystatin.

**Figure 3:**
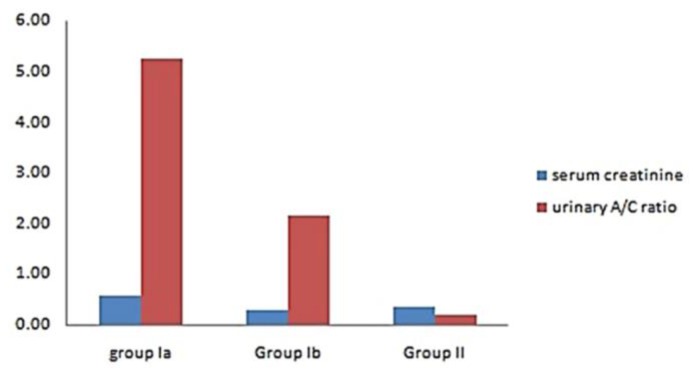
Comparison between studied groups regarding serum creatinine and A/C ratio.

During the acute hemolytic episode (group Ia), calculated GFR significantly decreased compared with normal healthy children (*p*=0.001) but no significant change when compared after relieving from the hemolytic episode (*p*=0.3) (Table 1, Figure 1). Serum cystatin C significantly decreased after the subsidence of hemolytic process (group Ib) compared with those during acute hemolysis (group Ia; *p*<0.001) and still elevated in those children after the subsidence of hemolysis (group Ib) compared with controls (group II; *p*=0.008) (Table 1, Figure 2). Urinary A/C ratio significantly increased during acute hemolysis and also after the elapse of the hemolytic process compared with the controls (*p*=0.001 for all) (Table 1, Figure 3).

No significant correlation between GFR and both reticulocyte count and hemoglobin level in group I patients either during acute hemolytic (*p*= 0.36, *r*=−0.1 for both reticulocyte count and Hb) episode and after the subsidence of the acute hemolytic crises. (for reticulocyte count *p*=0.79, *r*= −0.1 and for Hb, *p*=0.39, r=−0.16). (Table 2). Significant positive correlations between G6PD enzyme level and cystatin-based GFR in group Ia and Ib patients (*p*= 0.001, 0.001and *r*= 0.83, 0.65 respectively) (Table 2). Serum cystatin C levels significantly decreased after the subsidence of hemolytic process compared with those during acute hemolysis (*p*<0.001) and remained elevated in those children after the subsidence of hemolysis compared with controls (*p*=0.008) (Table 3, Figure 2). Urinary A/C ratio was elevated in group Ia and significantly reduced in group Ib (*p*<0.001) and still elevated in group Ib compared with controls (*p*=0.002) (Table 1, Figure 3).

## Discussion

Acute hemolytic anemia is associated with a significant burden on different tissues including kidneys. The incidence of acute kidney injury (AKI) related to hemolysis is not well described, but may be as high as 50% with massive hemolysis.^19^,^20^ and considered as one of the dangerous complications of severe hemolysis.^21^ It has been reported to occur in severe hemolytic episodes in G6PD-deficient subjects.^22^

In the present study, serum cystatin C and A/C ratio were measured, and GFR was calculated during the acute hemolytic episode and after the acute hemolytic crises had subsided.

During acute hemolytic crises*,* significant differences between children and controls regarding serum cystatin, A/C ratio, and GFR reflecting glomerular function depression during this acute stage.

This reduction in the glomerular function could be due to the free hemoglobin in the plasma^23^ and iron overload leading to massive hemosiderin deposition with their toxic effect in the proximal tubules and renal cortex. A second possibility is the micro medullary infarctions resulting from anemic hypoxia or inability of the hemosiderin-laden renal tubular epithelium to sustain maximum osmotic gradient between the urine and plasma. Severe anemia and hemoglobinuria-induce acute tubular necrosis due to tissue hypoxia and ischemia, as suggested by the metabolic acidosis at presentation, may be an alternative explanation. In children, however, this complication occurs rarely.^24^,^25^

The result of this cortical and tubular pathology is the impaired renal filtering functions and impaired creatinine excretion.^26^

No previous studies to assess serum cystatin in G6PD deficiency hemolytic anemia had been reported. Serum cystatin and creatinine levels were found to be increased in children with acute kidney injury.^27^

Serum cystatin C significantly decreased compared with their levels during acute hemolytic crises but still significantly increased compared with their levels in control group suggesting the incidence of a variable degree of kidney injury during the previous episodes of acute hemolytic crises. After fading from acute hemolytic crises and the blood, the picture should be normalized or near normalized, the reduced renal function seemly to be short lived. Some evidence of slow turnover of the renal hemosiderin.^28^,^29^ could explain the persistent some impairment of glomerular function after fading of acute hemolytic crises. G6PD activity per see can cause deleterious changes in cellular functions.^3^ An experimental study proved that the G6PD enzyme deficient mouse model is stimulating an inflammatory reaction in the kidney leading to an alteration in metabolic processing of albumin by the kidney tubules.^30^ G6PD, which is an indispensable component of antioxidant defense, plays pathogenic roles in diseases other than hemolytic disorders and may play a major role in the pathogenesis of the unexplained kidney diseases.^4^

Recovery phase after acute kidney injury occurs where the tubular function is restored and characterized by an increase in urine volume and a gradual decrease in BUN and serum creatinine to their pre-injury levels.^26^

Serum cystatin C level at the follow-up was significantly elevated compared to controls suggesting occult renal injury in these children. This datum is against the report of Krawczeski et al., 2010 who found that the optimal cut-off time at post burn was the day 14 (a condition produces the same drawbacks on the kidney as the acute hemolysis do) when both serum creatinine and serum cystatin were within normal range.^31^ This could be explained by the flow phase (following acute stages of burn-induced kidney injury) which is characterized by hyperdynamic circulation and hypermetabolic state. However, further studies are needed to validate this finding fully.

## Conclusions

G6PD deficiency is associated with a variable degree of occult kidney injury during acute hemolytic episodes. The renal injury may persist after the subsidence of the acute hemolytic episode. Further studies on a wide scale of patients with serial glomerular filtration rate evaluation over an extended period are needed to support our results.

## Figures and Tables

**Table 1:** Comparison between studied groups regarding some clinical and laboratory data.

Parameter		Group Ia N=30	Group Ib N=30	Group II N=30	P value			
					Group Ia&II	Group Ib&II	Group Ia&Ib	All groups
**Age (month)**	Range	5–96		2–95	0.06	0.09	0.84	0.12
	Median	24		36				
**Sex**	Male	18 (60%)		18(60%)	0.57	0.89	0.43	0.82
	Female	12 (40%)		12(40%)				
**Weight centile**	Range	5–75		5–75	0.11	0.38	0.17	0.26
	Median	27.5		27.5				
**Height centile**	Range	5–90		5–95	0.25	0.27	0.14	0.33
	Median	25		31				
**Hemoglobin level**	Range	2.2–7.1	10–14	9.8–13	<0.001*	0.09	<0.001*	<0.001*
	Median	4	11.8	11				
**Reticulocytic count**	Range	4–20	0.7–3.2	1–3	<0.001*	0.9	<0.001*	<0.001*
	Median	8	1.6	1.7				
**Indirect bilirubin**	Range	2.5–7.2	0.5–1.8	0.2–0.9	<0.001	0.008	0.001	<0.001
	Median	3.8	0.8	0.4				
**Serum cystatin C**	Range	0.41–1.64	0.38–1.1	0.36–.80	<0.001*	0.008*	<0.001*	<0.001*
	Median	0.91	0.66	0.50				
**Serum creatinine**	Range	0.40–0.90	0.1–0.5	0.1–.7	0.08	0.07	0.05	0.43
	Median	0.5	0.3	0.4				
**GFR**	Range	41.25–125	82.5–475	68–550	<0.001*	<0.001*	0.3	<0.001*
	Median	73.6	89.6	117.7				
**Urinary A/C ratio**	Range	1–19	0.1–5	0.2–0.4	<0.001*	0.002*	<0.001*	<0.001*
	Median	4.5	2	0.1				

BMI=body mass index, GFR= glomerular filtration rate, A/C ratio= albumin/creatinine ratio.

**Table 2:** Correlation between G6PD level and GFR in group I.

Variable		Group Ia	Group Ib
		GFR (ml/min/1.73m^2^)	
**G6PD (ng/ml)**	***p***	0.001*	0.001*
	***r***	0.83	0.65

G6PD= glucose six phosphate dehydrogenase, GFR= glomerular filtration rate.

**Table 3:** Correlation between GFR and some laboratory data.

Variable	GFR			
	Group Ia		Group Ib	
	*r*	*p*	*r*	*p*
**Reticulocytes (%)**	−0.17	0.36	−0.1	0.79
**Hb level (gm/dl)**	−0.17	0.36	−0.16	0.39
